# Debunking an old theory

**DOI:** 10.7554/eLife.62694

**Published:** 2020-10-16

**Authors:** Teresa Spix, Aryn Gittis

**Affiliations:** 1 Department of Biological Sciences, Carnegie Mellon UniversityPittsburghUnited States; 2 Neuroscience Institute, Carnegie Mellon UniversityPittsburghUnited States

**Keywords:** striatum, dopamine, Parkinson's disease, electrophysiology, Human

## Abstract

Recording the neural activity of cells in the brain of patients with Parkinson's disease challenges long-standing assumptions about how this disease manifests at the cellular level.

**Related research article** Valsky D, Heiman Grosberg S, Israel Z, Boraud T, Bergman H, Deffains M. 2020. What is the true discharge rate and pattern of the striatal projection neurons in Parkinson's disease and Dystonia?. *eLife* **9**:e57445. doi: 10.7554/eLife.57445

A group of structures deep inside the brain are thought to be responsible for the progression of Parkinson’s disease. These structures, known as the basal ganglia, play an important role in coordinating movement via two opposing motor pathways: the ‘indirect pathway’ which suppresses movement, and the ‘direct pathway’ which promotes movement. It has been reported that overactivation of the indirect pathway and underactivation of the direct pathway lead to the motor impairments associated with Parkinson’s disease (Albin et al., 1989; Bergman et al., 1990; Gerfen et al., 1990).

Previous studies investigating the cellular mechanisms that cause these abnormalities have largely focused on spiny projection neurons (SPNs for short), a group of cells found in a basal ganglia structure known as the striatum. These cells express one of two types of dopamine receptors called D1 and D2. SPNs expressing D1 are frequently referred to as the origin of the direct pathway, whereas SPNs expressing D2 are referred to as the origin of the indirect pathway. These pathways then pass this signal between multiple structures of the basal ganglia until they reach a group of cells known as the output nuclei.

The striatum receives most of its dopamine from an area in the brain that degenerates in Parkinson’s disease. This loss in dopamine is thought to reduce the activity of D1-SPNs and increase the activity of D2-SPNs, causing neurons in the striatum to fire at different rates: this is predicted to drive the excessive activity of the indirect pathway and reduced activity of the direct pathway, which leads to pathological activity throughout the basal ganglia (Gerfen and Surmeier, 2011; Figure 1A). This hypothesis is known as the ‘rate model’ and has had a huge influence on the field of Parkinson’s disease. Yet, there has been limited in vivo evidence showing the firing rates of SPNs changing, particularly from human patients.

**Figure 1.**
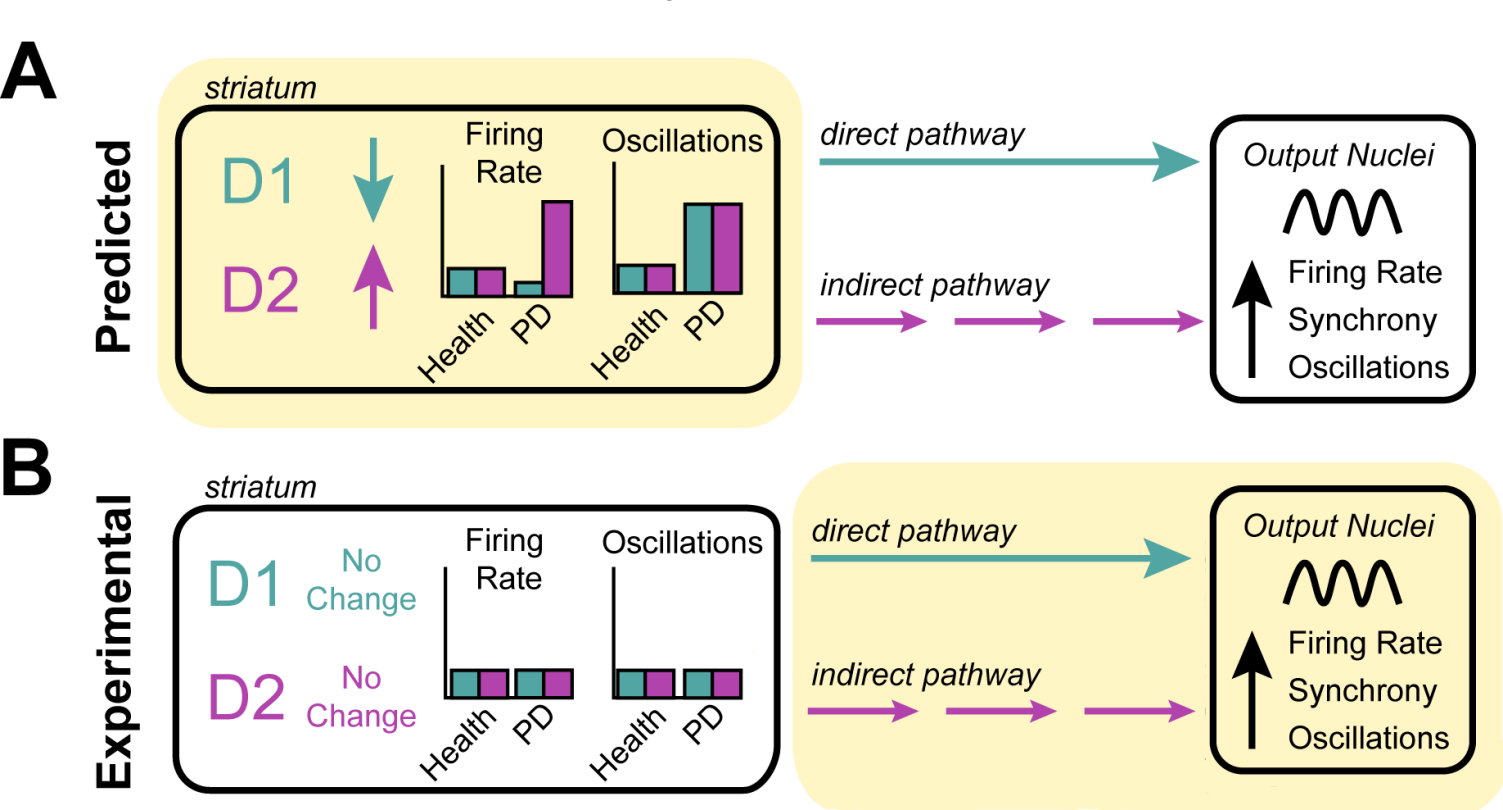
Investigating the origin of cellular features associated with Parkinson’s disease. (**A**) Neurons in the striatum express the D1 and D2 receptors for the neurotransmitter dopamine. The rate model predicts that the loss of dopamine in Parkinson’s disease decreases the activity of cells expressing the D1 receptor, increases the activity of cells expressing the D2 receptor (left), and increases the oscillatory activity of both D1 and D2 expressing neurons (right). These changes in activity are thought to alter the direct (turquoise) and indirect (purple) signals that D1-neurons and D2-neurons send to other structures in the basal ganglia. It is thought that this causes downstream neurons in the output nuclei of the basal ganglia to fire faster, more synchronously and with more oscillations – the pathophysiology commonly found in patients with Parkinson’s disease. (**B**) Valsky et al. tested this model on human patients with Parkinson’s disease and could not find any evidence of neurons in the striatum changing their firing rates or patterns of activity. This suggests that the neurological features associated with Parkinson’s disease do not stem from the striatum (as predicted by the rate model) but instead may originate downstream from the striatum (highlighted in yellow), in other structures of the basal ganglia.

Image credit: Teresa Spix.

Now, in eLife, Marc Deffains (University of Bordeaux) and colleagues – including Dan Valsky (Hebrew University of Jerusalem) as first author – report experiments investigating the firing rates of SPNs in patients with Parkinson’s disease (Valsky et al., 2020). The team were able to gather data from human patients who were undergoing a surgical procedure that implants electrodes into deep regions of the brain. Valsky et al. found that the firing rates of SPNs in patients with Parkinson’s disease were no different than expected values found in healthy non-human primates. Further computational analysis, clustering the different firing rates detected, was unable to identify two distinct populations of neurons that could represent overactive D2-SPNs and underactive D1-SPNs.

These results were in contrast to the only other human study which supports the predictions made by the rate model (Singh et al., 2016). However, both studies used a different method to isolate and analyze the activity of neurons. Valsky et al. applied strict criteria to ensure that the firing rates recorded only came from well-isolated, stationary single units. This minimizes the chance of other factors, such as noise from movement or signals from damaged cells, interfering with the firing rates being measured. Valsky et al. showed that when these criteria were not in place, they were able to replicate the changes in activity reported in the previous study, but argued that this is a spurious conclusion.

The fact that Valsky et al. were not able to find evidence for the rate model within the striatum was not entirely unexpected, as the exceptions and limitations of this model have become increasingly documented (Obeso and Lanciego, 2011). Other models have proposed that the abnormalities observed in the basal ganglia arise from neurons changing their patterns of activity to fire more irregularly or with increased oscillations (Nelson and Kreitzer, 2014). But when Valsky et al. searched their data for these other patterns of activity, they could not detect any of these features in the striatal neurons of patients with Parkinson’s disease (Figure 1B).

These findings raise a lot of questions about the role the striatum plays in the motor impairments associated with Parkinson’s disease. However, two critical variables that were not addressed in this study are synchrony (non-oscillatory), and total neuron recruitment within the striatum. When multiple SPNs fire simultaneously, this relays a more powerful signal to downstream areas of the brain than if SPNs are activated independently. Therefore, if dopamine depletion enabled more D2-SPNs to activate simultaneously, or in greater numbers, this could lead to increase indirect pathway output from the striatum. However, the techniques used to record neuronal activity in this study means it is not possible to determine whether this change occurred.

These findings highlight the need for a critical reassessment of long-standing assumptions about the cellular mechanisms involved in the onset of Parkinson’s disease. Even if this study does not completely rule out the striatum as a source of basal ganglia abnormalities in this condition, it narrows down the types of changes that might be responsible. It also emphasizes the roles of brain structures that are traditionally considered to be ‘downstream’ of the striatum in generating the neuronal defects associated with Parkinson’s disease (Figure 1B). Furthermore, this work provides a valuable, rigorously curated data set which will be beneficial to the field.
